# Cardiovascular risk profile in two cohorts of young apparently healthy South Asian descendants in the Netherlands: still a long way to go!

**DOI:** 10.1097/MCA.0000000000001364

**Published:** 2024-04-25

**Authors:** Sanjay N. Gobardhan, Pranobe V. Oemrawsingh, Su S. Liem, Suzanne C. Cannegieter, Martin J. Schalij

**Affiliations:** a Department of Cardiology, Leiden University Medical Center, Leiden; b Department of Cardiology, Medical Center Haaglanden, The Hague; c Department of Cardiology, Amstelland Hospital, Amstelveen; d Department of Clinical Epidemiology, Leiden University Medical Center, Leiden, The Netherlands

**Keywords:** cardiovascular risk, coronary artery disease, Framingham risk score, South Asians

## Abstract

**Background:**

Cardiovascular disease (CVD) imposes a major healthcare burden on young descendants of South Asian migrants living in the western world. In comparison to the native population, the prevalence is significantly higher and the prevalence of CVD risk factors is increasing rapidly. The cardiovascular risk profile and 10-year risk scores of South Asian descendants were evaluated in two cohorts with a 10-year time difference.

**Methods:**

Two cross-sectional studies, conducted in 2004 and 2014, focused on asymptomatic South Asian descendants aged 18–59 years were performed. A short questionnaire, BMI, waist circumference, blood pressure, and nonfasting blood tests were obtained. The cohort of 2014 was matched with the cohort of 2004, based on age, gender, and family history of CVD.

**Results:**

In 2014, 674 South Asians (44% men, age 38.2 ± 12.0 years) were matched with 674 South Asians (44% men, age 38.3 ± 12.1 years) included in 2004. Notably, hypertension prevalence decreased significantly in 2014 (10.6% vs 23.1% in 2004, *P* < 0.001), while mean BMI increased (26.1 vs 24.9, *P* < 0.001). The mean Framingham risk score was lower in 2014 (5.31 ± 6.19%) than in 2004 (6.45 ± 8.02%, *P* < 0.05).

**Conclusion:**

This study demonstrates that South Asian descendants in 2014 have a lower but still high absolute risk for coronary events compared to 2004. Important differences in cardiovascular risk profile exist. Despite improvements, South Asian descendants in 2014 still face a high absolute risk for coronary events compared to 2004, indicating the necessity for continued primary prevention and lifestyle interventions.

## Introduction

Cardiovascular disease (CVD) is a major healthcare burden in young descendants of South Asian migrants living in the western world. Numerous studies demonstrated a 3–5 times higher CVD prevalence in this group in comparison to the native Caucasian population [1–4]. Furthermore, the prevalence of CVD risk factors is also increasing rapidly in this South Asian population. Prevalence of hypertension, glucose intolerance, atherogenic dyslipidemia, thrombotic tendency, subclinical inflammation, and endothelial dysfunction are disproportionately higher in this ethnic group, despite a lower BMI [5–7].

There might be a racial genetic predisposition to developing CVD, but so far the mechanisms are not well understood [8]. Furthermore, there is still no definitive evidence that a South Asian origin can be used as an independent risk factor when considering a patient’s CVD risk in order to plan interventions. South Asians, however, have higher rates and earlier onset of coronary artery disease due to predisposition and lifestyle factors [9].

In the Netherlands, a large South Asian community of approximately 250 000 persons lives. This community mainly consists of migrants from Surinam who migrated after the independence of Surinam in the seventies of the previous century to the Netherlands. Their ancestors moved from Bihar, India to Surinam in 1863 after the abolishment of slavery. The current community forms the third to seventh generation of South Asians. As in other western countries, this group of young generation South Asian descendants exhibit higher numbers of early onset cardiovascular morbidity and mortality in comparison with the native Caucasian population [10,11].

To evaluate the cardiovascular risk profile (and the development over time) in South Asian descendants in the Netherlands, the first part of the SHIVA study (Screening of HIndustans for cardioVAscular risk factors) was performed in 2004. The group studied in 2004 demonstrated the persistence of an unfavorable cardiovascular risk profile in young, third to seventh generation migrated South Asians and supported an aggressive screening and intervention strategy [12].

The second part of the SHIVA study was performed in 2014 with the objective to reassess the prevalence of CVD risk factors and the cardiovascular risk scores of asymptomatic third to seventh generation South Asian descendants and to compare the risk profile with the 2004 group.

## Methods

### Study design

Two cross-sectional population based studies were performed to compare the cardiovascular risk profile and 10-year cardiovascular risk scores of two cohorts of third to seventh generation South Asian descendants of whom their ancestors moved from the Bihar, India to Surinam in 1863 after the abolishment of slavery and later to the Netherlands.

### Study population

South Asian participants (18–60 years) were recruited in July 2004 and July 2014 at the Milan Cultural Festival held in The Hague, the Netherlands. This festival is organized annually and attended by 70 000 to 80 000 South Asians.

In this study, South Asians descendants are defined as people originating from the South Asian subcontinent which contains the countries: India, Pakistan, Sri Lanka, Maldives, Nepal, Bhutan, and Bangladesh. Currently South Asians cover one fifth of the global population.

To be eligible for the SHIVA study, participants had to self-identify as South Asian ethnicity (at least one parent’s ancestor originated from the Indian subcontinent). South Asians born in the Indian subcontinent were excluded. Subjects were classified as asymptomatic when they did not have documented diabetes, hypercholesterolemia, hypertension, or ischemic heart disease and were currently not receiving any form of treatment for any of these conditions. All others were excluded.

In 2004, 1790 South Asians were included in the analysis [12]. In 2014, 931 participants were screened, of whom 257 subjects (27%) were excluded; 39 due to unknown or neither South Asian nor Dutch origin; 145 participants for not being asymptomatic; 21 because of unknown place of birth or born in the Indian subcontinent; 116 subjects who did not match the age range of interest (18–59 years). In total 674 participants remained in the 2014 study.

To compare the two groups, and to improve the comparability of groups and reduce the potential for confounding variables, participants in 2014 were matched by age, gender, and prevalence of family history of CVD with 674 participants out of the 1790 subjects from the 2004 cohort. In total 1348 participants were included in the analysis. Even though there is a relatively small sample size, the sample size is good enough to describe characteristics, behaviors, or phenomena within this matched population. Since descriptive studies do not involve hypothesis testing or statistical inference, considerations such as statistical power is less relevant.

### Survey methods

In both studies similar procedures were performed. Demographic, self-reported behavioral information (smoking, alcohol, and physical activity), objective measures of anthropometry and biochemical measures were collected. History of any chronic illness, in the participant as well as in the family, including diabetes, hypertension, hypercholesterolemia, cerebrovascular accident, and CVD was recorded.

### Ethical approval

The SHIVA-study was approved by the Ethics Committee of the Leiden University Medical Center. The procedure was explained to all participants. All participants provided written informed consent.

### Anthropometric profile

Standardized anthropometric measurements, including height, weight, and waist circumference, were obtained by trained volunteers. Waist circumference was measured above the iliac crest and below the ribs. A waist circumference of ≥90 cm for men and ≥80 cm for women was considered as abdominal obesity [13]. BMI was calculated as weight in kilograms over height in meters squared, overweight was defined as a BMI ≥ 23 kg/m^2^ and obesity as BMI ≥ 27 kg/m^2^. According to the WHO, the cutoff value that defines obesity in South Asians is lower than that for whites [14]. One-off blood pressure was measured by trained nurses using an automated blood pressure monitor (Omron M7 comfort, Omron Healthcare Europe BV, Hoofddorp, The Netherlands). Blood pressure was recorded in a sitting position with an appropriately sized cuff applied to the upper arm and could only be performed once instead of serially. Hypertension was defined with the commonly definition of hypertension (systolic blood pressure ≥ 140 mmHg or a diastolic blood pressure ≥ 90 mmHg) [15].

### Biochemical analysis

Nonfasting blood samples (35 μl) were obtained for the estimation of blood glucose and lipid profile, using the Cholestech LDX analyzer (Cholestech Corporation, Hayward, California, USA). Glucose, total cholesterol (TC), triglycerides, high-density lipoprotein cholesterol (HDL-C), and low-density lipoprotein cholesterol (LDL-C) were calculated via the Friedewald formula [16]. All tests were administered by trained nurses. LDL-C ≥2.5 mmol/l indicated high levels, while HDL-C ≤ 0.9 mmol/l indicated low levels. Nonfasting glucose levels of ≥7.7 mmol/l and ≤11 mmol/l indicated impaired glucose tolerance, while >11 mmol/l indicated diabetes [17].

### Framingham risk score

The 10-year risk of CVD for an individual participant was estimated by applying the cardiovascular risk equation based on the Framingham Heart Study [18]. These are derived multivariable mathematical functions that assign weights to risk factors such as: gender, age, blood pressure, TC, HDL-C, smoking behavior, and prevalence of type 2 diabetes. This risk score was applicated, due to the quick assessment during screening and was also used in the SHIVA study in 2004 [12].

### Data collection

All data were entered into a customized database and the 10-year Framingham risk scores (FRS) were calculated [19]. Every participant received a printed copy of their risk assessment and a personal lifestyle recommendation.

### Data analysis

Prevalence and mean of each risk factor were calculated for both matched groups. To examine differences between the two groups in continuous and categorical variables, independent Student’s *t*-test, one-way analysis of variance or Mann–Whitney *U* test, were applied. To identify potentially modifiable risk factors in each age group, we assessed the prevalence of risk factors in each 10-year age group.

Individual estimated FRS of the South Asians were categorized in as 0–5, >5, >10, >15, and >20% and also for each 10-years age group. All statistical analyses were performed using IBM SPSS v 29.0.0; *P*-values (two sided) of 0.05 or less were regarded as significant.

## Results

### Population characteristics

In this analysis, 1348 South Asian descendants were recruited. Due to the matching, the groups were similar with respect to age (38.3 ± 12.0 years for the 2014 group; age 38.2 ± 12.1 years for the 2004 group (NS)), gender (43.9% men in 2004 and 2014), and prevalence of family history of CVD (29.8% in 2004 and 2014).

### Prevalence of cardiovascular risk factors

Cardiovascular risk factors and blood chemistry measurements for both groups are shown in Table 1.

**Table 1:** Cardiovascular risk factors of South Asian descendants in the Netherlands group by year

	South Asians 2004, *n* = 674	South Asians 2014, *n* = 674	*P*-value
Age (years)	38.2 ± 12.0	38.3 ± 12.1	Matched
Men (sex), *n* (%)	296 (43.9)	296 (43.9)	Matched
Family history of			
Cardiovascular disease, *n* (%)	201 (29.8)	201 (29.8)	Matched
Hypertension, *n* (%)	311 (46.1)	353 (52.4)	0.02
Hyperlipidemia, *n* (%)	109 (16.2)	206 (30.6)	<0.001
Diabetes, *n* (%)	338 (50.1)	347 (51.5)	0.62
Smokers, *n* (%)	127 (18.8)	142 (21.1)	0.31
Alcohol intake, *n* (%)	198 (50.1)	259 (38.0)	<0.001
Waist circumference (cm)	89.4 ± 11.2	91.8 ± 11.8	<0.001
>90 cm for men or >80 cm for women, *n* (%)	445 (66.0)	486 (72.1)	0.011
BMI (kg/m^2^)	24.9 ± 3.9	26.1 ± 4.6	<0.001
BMI ≥ 23 kg/m^2^, *n* (%)	455 (67.5)	487 (72.2)	0.015
BMI ≥ 27 kg/m^2^, *n* (%)	188 (27.9)	252 (37.5)	<0.001
Systolic blood pressure (mmHg)	133.7 ± 19.2	125.0 ± 17.2	<0.001
Diastolic blood pressure (mmHg)	80.2 ± 11.5	79.3 ± 11.5	0.16
SBP ≥ 140 mmHg or DBP ≥ 90 mmHg, *n* (%)	258 (38.5)	162 (24.1)	<0.001
Cholesterol (mmol/l)	4.99 ± 0.95	4.90 ± 0.92	0.11
High density lipoprotein (HDL) cholesterol (mmol/l)	1.16 ± 0.32	1.18 ± 0.36	0.20
≤0.9 mmol/l, *n* (%)	139 (20.7)	154 (23.1)	0.29
Low density lipoprotein (LDL) cholesterol (mmol/l)	2.86 ± 0.84	2.81 ± 0.75	0.70
≥2.5 mmol/l, *n* (%)	438 (65.0)	390 (57.9)	0.27
Triglycerides (mmol/l)	2.19 ± 1.43	2.04 ± 1.26	0.04
Total cholesterol/HDL ratio	4.64 ± 1.60	4.48 ± 1.55	0.06
>5, *n* (%)	230 (34.3)	197 (29.9)	0.09
Nonfasting glucose (mmol/l)	5.84 ± 1.40	6.03 ± 1.19	0.009
≥7.8 to ≤11 mmol/l, *n* (%)	39 (5.8)	43 (6.4)	0.65
>11 mmol/l, *n* (%)	6 (0.9)	3 (0.4)	0.32
10-years Framingham risk score (%)	6.45 ± 8.02	5.31 ± 6.19	0.022

Values are expressed as mean ± SD or number (%).

South Asians in 2014 had a significantly higher prevalence of family history of hypertension (52.4 vs 46.1%) and hyperlipidemia (30.6 vs 16.2%) compared with the South Asians in 2004.

Smoking was slightly, but not significantly, more prevalent in 2014 (21.1%) compared to 2004 (18.8%). Fewer South Asians consumed alcohol in 2014, 38.0 vs 50.1% in 2004. In 2014 the prevalence of obesity and central obesity was significantly higher compared with the 2004 group (respectively, 17.2 vs 8.3% and 39.6 vs 29.4%). The mean systolic blood pressure (133.7 mmHg in 2004 vs 125.0 mmHg in 2014) and the overall prevalence of hypertension (23.1% in 2004 vs 10.6% in 2014) was, however, lower in 2014. Small differences in lipid profiles were observed. HDL-C was similar in both groups. Mean LDL in 2014 was 2.81 mmol/l compared to 2.2.86 in 2004 (NS). Mean nonfasting glucose was significantly higher in 2014, that is, 5.84 mmol/l in 2004 vs 6.03 mmol/l in 2014. Impaired glucose tolerance, however, was not different between the two groups. Among the South Asians 0.9% in 2004 and 0.4% in 2014 were diagnosed as de novo diabetes.

### Prevalence of modifiable risk factors by age group

**Fig. 1:**
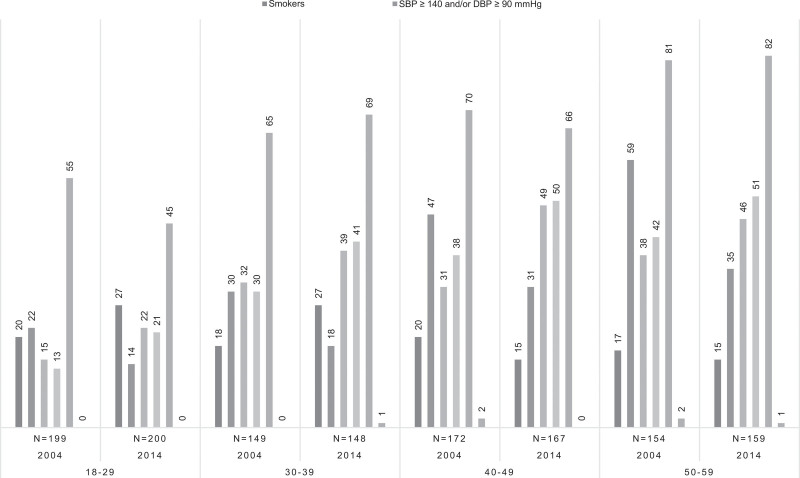
Modifiable risk factors in South Asian descendants group by age group and year. Values are expressed in %. Central obesity = BMI ≥ 27 kg/m^2^; abdominal obesity = waist circumference >90 cm for men or 80 cm for women. DBP, diastolic blood pressure; LDL, low density lipoprotein; SBP, systolic blood pressure.

Figure 1 shows the prevalence of modifiable risk factors grouped by age groups. In contrast with 2004, central and abdominal obesity was one of the most common abnormalities in all age groups in 2014. Remarkably, in all age groups of 2004 and 2014, a high prevalence (>45%) of high nonfasting LDL (>2.5 mmol/l) existed.

### Framingham risk scores

The mean cardiovascular risk among the South Asian population in the Netherlands in 2004 was 6.45% which was significantly higher compared to 5.31% in 2014 (Table 1).

**Fig. 2:**
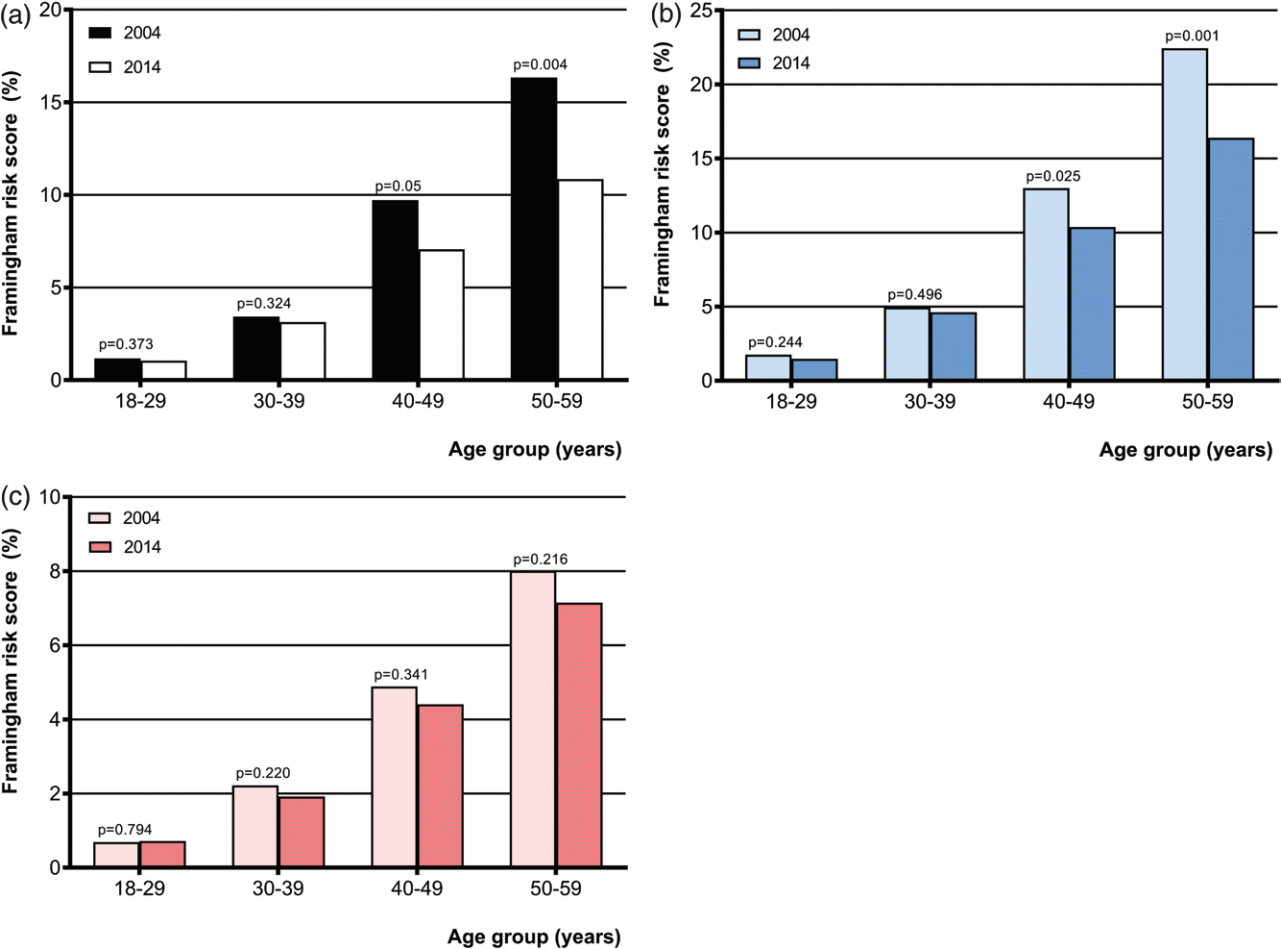
Cardiovascular risk in South Asian (a) descendants, (b) men, and (c) women in the Netherlands in 2004 and 2014 group by 10-year age group.

Figure 2a shows South Asians aged 40–59 in 2014 had a significant lower risk score compared to the 2004 group. The 2014 South Asian men aged 40–59 years exhibited a significantly lower FRS (Fig. 2b). No significant differences between the two groups, however, were observed in the FRS group per 10 year-age groups in South Asian women (Fig. 2c).

**Fig. 3:**
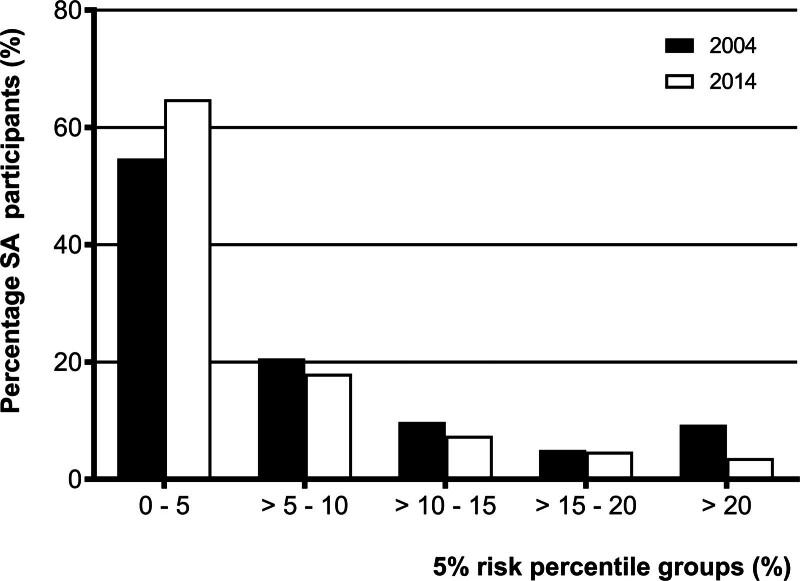
Framingham risk score in South Asian descendants in the Netherlands group by 5% risk percentile.

Figure 3 displays the FRS group per 5% risk percentile. The 2014 South Asian descendants had a higher percentage of participants in the 0–5% cardiovascular risk group and a lower percentage of participants in the >5% cardiovascular risk groups than the South Asian participants in 2004.

## Discussion

Key findings of this study are:

(a) The cardiovascular risk of both groups of South Asian descendants is still very high. (b) Despite the known risk it is striking to notice the high prevalence of modifiable risk factors in each age group (and regardless of gender). (c) Notwithstanding the already elevated CVD risk profile even more participants were smoking in 2014 than in 2004. (d) Before the age of 30 years multiple modifiable risk factors were present in a large number of South Asian descendants in both groups, however, a small but positive change is noted in the 2014 group.

This cross-sectional study with adequate statistical power and representativeness (*n* = 1348) was conducted for the first time in 2004 and repeated in 2014 among an apparently healthy South Asian population in the Netherlands. Strengths of this study include a large population-based sample, representative sampling methodology, and assessment of multiple cardiovascular risk factors.

### Prevalence of cardiovascular risk factors

Multiple studies demonstrated that South Asians have a 3–5 times increased risk for CVD compared to other ethnic population groups [20–22]. In general, myocardial infarction develops 5–10 years earlier in South Asians than in other ethnic population [23].

Nevertheless, several studies reported no higher prevalence of conventional risk factors such as smoking, hypertension, and hypercholesterolemia in South Asians than in other ethnic groups. Other atherosclerotic risk factors, however, are particularly prevalent in South Asians, including increased level of TC to HDL ratio, high triglycerides, type 2 diabetes, and central or visceral obesity [24,25]. High prevalence of obesity among South Asians was reported in earlier studies and is associated with a high prevalence of hypertension, diabetes, and dyslipidemia [26].

In 2009, Liem *et al*. conducted a cross-sectional study comparing the prevalence of cardiovascular risk factors among 1790 South Asians (45% men, age 35.9 ± 10.7 years) and 370 native Dutch Caucasians (23% men, age 40.8 ± 10.1 years) as control group [12].

This initial phase of the SHIVA study was conducted, employing identical standardized anthropometric measurements, biochemical analyses, and risk score interpretation methodologies as those utilized in our second phased study. Revealing higher rates of positive family history for CVD, hypertension, and diabetes among South Asians. They also exhibited elevated levels of TC, VLDL, and triglycerides, along with lower HDL levels and increased glucose intolerance. Overweight and central obesity were more prevalent in South Asians, particularly among women. Notably, insulin resistance was common in South Asian men even without excessive body fat or abdominal obesity. In conclusion, prevalence of most of the conventional and modifiable cardiovascular risk factors in each 10-year age group was higher in South Asians compared with controls, which reflected in higher FRS.

The HELIUS study in the Netherlands is one of the pioneering investigations shedding light on health disparities among diverse ethnic groups, notably South Asians and Dutch Caucasians, in the Netherlands. Hence, in 2008, Bindraban *et al*. conducted a cross-sectional study among multiple ethnicities, under which 336 South Asians and 486 Dutch Caucasians, aged 35–60 years to develop a new risk score for diabetes [27]. Population characteristics showed a higher prevalence of family history of CVD, higher BMI, higher waist circumference, lower HDL-levels, and higher blood pressures in the South Asian ethnicity group. In 2018, Perini *et al*. disseminated a manuscript investigating ethnic differences in metabolic cardiovascular risk among normal weight individuals (BMI ≤ 25 kg/m^2^), notably 491 South Asians and 1017 Dutch Caucasians, from the HELIUS study [28]. Remarkably, despite maintaining a BMI ≤ 25 kg/m^2^, South Asians exhibited a higher prevalence of abdominal obesity and a greater incidence of familial CVD history compared to their Dutch Caucasian counterparts.

In South Asians, conventional cutoff limits for BMI might not define overweight and obesity correctly because of their higher percentage of body fat and less mass as compared with Caucasians [29]. Some studies reported lower BMI cutoffs for South Asians based on body fat as standard [30,31].

In line with these observations, in this study the 2014 group showed a significantly higher BMI and waist circumference as compared to the 2004 group. The higher BMI and larger waist circumference were noticed globally during the last decade [32]. Conversely nonsignificantly lower TC and higher HDL levels were measured in 2014 compared to 2004. Alcohol consumption was more common in South Asians in 2004 compared to 2014. It is known that alcohol drinking affects blood pressure, HDL levels, and triglycerides [33]. TC and HDL levels can also be influenced (positively) by exercise and healthier eating. Elevated blood pressure and dyslipidemia were less prevalent in 2014 compared to 2004. Smoking, however, is still an important issue to address in 2014. Dyslipidemia was common in most age groups in 2004 and 2014, similar findings were reported in other studies among South Asians [34,35].

### Prevalence of modifiable risk factors by age group

The higher prevalence of cardiovascular risk factors in younger age groups in the studied population is of particular concern. Before the age of 30 years multiple modifiable risk factors were already present in a large number of South Asian descendants in the Netherlands in 2004 and 2014. Prevention strategies therefore should focus on the younger age groups, main targets should be to revert conditions as smoking, dyslipidemia, overweight.

### Framingham risk scores

The Framingham risk assessment was used to estimate the 10-year CVD risk in South Asians. This assessment was designed to estimate cardiovascular risk in Caucasians. Several studies concluded that the Framingham model, however, predicts CVD outcomes fairly well in South Asians [36]. Other studies have shown that the risk assessment models developed for western populations systematically underestimate risk in South Asian descendants [37,38]. Therefore, it is still unknown which cardiovascular risk score estimation is the best to use in South Asian descendants in the western world.

In this study, South Asians in 2004 exhibited higher 10-year FRS compared with South Asians in 2014, unfortunately, the absolute risk is still high in both groups. Of interest, the risk in men dropped significantly between 2004 and 2014 whereas it remained the same in women (despite the significantly lower risk score compared to men in both groups).

The consistent high risk scores, both in men and women, found in 2004 and 2014 justify an aggressive screening and treatment strategy which should start at a relatively young age.

### Effectiveness of prevention programs

Several epidemiological studies showed that physical inactivity increases cardiovascular risk [39,40]. Lifestyle improvement programs are mandatory and should focus on lifestyle, exercise, and nutrition. If necessary additional drug therapy should be used [41].

The SHIVA screening in 2004 assessed high awareness among South Asians and healthcare providers, mainly general practitioners, in the Netherlands. Therefore, a positive change was expected in cardiovascular risk profile in 2014. In 2014 mean systolic blood pressure and the prevalence of hypertension were lower than in 2004. A speculative explanation of the lower prevalence of hypertension could be nonpharmacological prevention. Particularly, reduction of sodium intake and adequate potassium intake by increasing the amount of fruits and vegetables. A small but positive change has been noted in the lipid profiles of South Asian descendants in the Netherlands. Diet programs and reduction of use of unsaturated fatty acids are globally promoted and used more. Aggressive primary prevention programs, however, should be assessed more, especially in South Asian men.

### Limitations

This study has some limitations which should be discussed. First, our study is based on cross-sectional self-reported data, this could have biased the results of behavioral factors. Second, selection bias was possible because only at the Milan Festival in The Hague patients were asked to participate in this study. There was, however, no indication that the included group differed importantly from the general South Asians population living in the Netherlands. South Asians from all regions in the Netherlands visit the yearly Milan Festival in The Hague. Remarkably, in 2014 a large group young South Asian descendants aged 30–39 years were already screened or treated by a doctor and therefore excluded for participation. This was a difference compared to the 2004 group. General practitioners and cardiologists are aware of the unfavorable cardiovascular risk profile of South Asian descendants in the Netherlands.

Third, due to the setting, nonfasting blood samples were used for biochemical analysis in most participants. This affected the LDL, triglycerides, and glucose measurements. An under- or overestimation of the biochemical analysis might have occurred. In addition, automatic blood pressure measurements were performed only once instead of serially. Overestimation of prevalence of hypertension might therefore have occurred. Therefore, the defined blood pressure was also noted with a little higher systolic blood pressure than normally. Instead of a systolic blood pressure of 140 mmHg, we defined a systolic blood pressure of 160 mmHg as hypertension. Fifth, no comparative data with a control group of Dutch Caucasians was employed in this study. This decision was made as the primary aim of the study was to compare and assess cardiovascular risk profiles between two generations of young South Asian individuals. Consequently, we referenced three studies that conducted similar comparisons. Lastly, the Framingham risk assessment was used to predict cardiovascular risk in South Asian participants. This assessment was used in 2004, therefore also in 2014. Risk factors as obesity, family history of CVD, and elevated triglyceride levels are common in our study population, but not included in the Framingham risk assessment. In daily clinical practice, this risk estimator is still the best guiding tool for cardiovascular risk management.

### Conclusion

This study demonstrates that South Asian descendants in 2014 have a significantly lower but still high absolute risk for coronary events compared to South Asians in 2004. Important differences in the cardiovascular risk profile exist. Despite some advances, primary prevention and lifestyle improvement programs, however, remain mandatory. Long-term follow-up of the 2004 cohort will help to define whether CVD risk prediction is similar among South Asians in comparison with other ethnic groups.

## Acknowledgements

The SHIVA study is being conducted by the Leiden University Medical Center and Medical Center the Hague. The authors thank staff at the Leiden University Medical Center and Medical Center the Hague for their collaboration and support in conducting this project.

### Conflicts of interest

There are no conflicts of interest.
